# Does Virtual Fencing Work for Grazing Dairy Cattle?

**DOI:** 10.3390/ani9070429

**Published:** 2019-07-08

**Authors:** Sabrina Lomax, Patricia Colusso, Cameron E.F. Clark

**Affiliations:** Livestock Welfare Group, School of Life and Environmental Sciences, Faculty of Science, University of Sydney, Camden, NSW 2570, Australia

**Keywords:** Virtual fence, dairy, grazing, associative learning, technology, behavior, paddock usage

## Abstract

**Simple Summary:**

Fences are used to prevent the over and under grazing of forages by herbivores. These fences can either be permanent, temporary or virtual. Virtual fencing uses collar-mounted GPS devices to contain animals within an area. The collars emit an audio tone as the animal approaches the virtual fence line. If the animal continues forward, an electrical pulse is applied. However, if the animal stops or turns around, they do not receive a pulse. We evaluated the application of virtual fencing for grazing dairy cows, to gain an understanding of how individuals learn virtual fence simuli. The virtual fence contained cattle within predetermined areas for most of the time (99%). However, there was significant variation between individuals for the number and type of interactions with the virtual fence, and animal location within the paddock varied. The success of maintaining animals within a grazing area may have costs for both individual animal welfare and efficient pasture utilization.

**Abstract:**

Pasture management in Australia’s dairy industry requires the manual shifiting of temporary electric fences to maintain pasture quality and growth. Virtual fencing presents an alternative to save time and labour costs. We used automated virtual fence (VF) collars to determine the variation in learning of the virtual fence stimuli, and evaluated the success of the technology to contain cows in a predetermined area of pasture. Twelve Holstein-Friesian non-lactating multiparous dairy cows were fitted with the collars, and a VF was used to restrict cows to two grazing allocations (G1 and G2) across six days. Cows received an audio tone (AT) when they approached the virtual fence, and a paired electrical pulse (EP) if they continued forward. The VF contained cows within predetermined areas for 99% of time, but cows spent the least time near the fence (*p* < 0.01). The number of stimuli reduced through time, demonstrating the ability of cows to learn the VF (*p* = 0.01). However, the mean number of EP per day ranged from 1 to 6.5 between individuals (*p* < 0.01). Therefore, successful containment may have a welfare cost for some individuals. Further work should focus on this individual variation, including measures of welfare.

## 1. Introduction

Almost all (95%) Australian and New Zealand dairy systems are pasture based [1]. Pasture management within these systems presents significant challenges associated with labour costs, time management and efficient feed allocation. Dairy systems predominantly rely on temporary electric fencing for flexible and efficient allocation of fresh pasture. While these electric fencing systems allow for the control of pasture allocation, they can be limiting, as they require manual labour to manage frequent changes in pasture allocation [2]. For more efficient pasture management, and to reduce soil damage, fences can be shifted several times per day, and require both a front and back fence to prevent grazing outside the allocated area. Holding dairy cows on previous pasture allocations or a fraction of that day’s allocation through strategic fence placement until all cows have arrived post-milking could be a potential strategy to overcome differences in pasture nutritive value consumed across a herd due to milking order [3]. A fence that moved across time to bring cattle back to the dairy would also save farmers considerable time both in conventional or automatic milking systems. In this regard, the remote, automatic placement of fences such as a ‘virtual fence’ would enable these benefits to be realised without additional labour costs. For pasture-based dairy systems, virtual fencing not only has the potential to improve the flexibility and efficiency of pasture allocation, but also allows for an improvement in the quality of life with reduced labour input [4].

A virtual fence (VF) can be defined as an enclosure, a barrier, or a boundary without a physical barrier [2]. Cattle can be successfully contained within a VF boundary without the use of ground-based fencing [5,6,7,8,9,10]. Early forms of the technology relied on electromagnetic coupling between a device on the animal, typically as a collar, and an induction cable either on or buried under the ground [2,4,11]. However, such fixed systems mirrored the limitations of those physical barrier fencing systems above ground.

Physical barriers or conventional electric fences work by the pairing of a visual stimulus with a sensory stimulus such as an electric stimuli. Through the pairing of stimuli to elicit a response, cattle learn to avoid the boundary, and the electric stimulus, by responding to the visual stimulus alone. Recent advances in virtual fence technology rely on this approach through the pairing of an audio tone with an electrical pulse [5]. Cattle have been successfully trained to avoid an area or feed attractant using these paired stimuli, delivered via collar-mounted devices [5,8,9,10,12]. Recent studies using an earlier prototype of these collars found that Angus heifers were able to learn VF stimuli to respond to multiple changes of a VF and remain within the relative inclusion zones over 20 days [9]. Similarly, cows were able to be excluded from a riparian zone for 10 days [13]. However, cattle trained to a VF barrier showed greater avoidance of the location where electrical stimuli were received than those trained to an electric fence [14]. This raises the question as to whether VF stimuli affect how cows use the space within a virtual paddock. The behaviour of cattle in a grazing environment can be independent to that of other herd members [15]. Therefore, it is important to quantify this spatial usage on an individual level. Research has demonstrated that Angus heifers are able to learn to respond to the audio tone alone to avoid a VF boundary. However, there is a high degree of variation between individuals in the rate of learning and the quantity of VF stimuli delivered [9,13]. There is limited information on how individuals within a herd learn and respond to VF stimuli through time. Traditionally, the effect of interventions has been determined at a group level, with limited focus on the individual [16]. However, understanding the variability in animal response to cues and controls within a herd is essential when implementing technology that will modify an animal’s behaviour, as there can be subsequent welfare issues associated with inability to learn [17].

Our experiment had three objectives. The first objective was to determine the proportion of time that dairy cattle were maintained on pasture behind a virtual fence. The second objective was to determine the spatial distribution of dairy cows within the grazing allocation relative to the virtual fence and, finally, to determine the variability in dairy cow learning and response VF stimuli.

## 2. Materials and Methods

This experiment was conducted between 26 September and 4 October 2018 at The University of Sydney’s research farm near Camden, NSW Australia. All procedures were approved by the University of Sydney’s Animal Ethics Committee (AEC Approval No. 2018/1306).

### 2.1. Animals and Management

Twelve Holstein–Friesian non-lactating, multiparous dairy cows were used (mean ± SD: 5 ± 1.4 years old; 666 ± 71 kg liveweight). All cattle were experienced with strip-grazing pasture, but naïve to virtual fence (VF) collars at the beginning of the study. Therefore, all cows were subjected to a period of habituation to the collars for 2 days (Days −2 to 0) before the experimental period. On day −2, the cows were moved to the cattle yards where they were weighed and restrained in the cattle crush for VF collar fitting. The collars were tightened so that they would remain on the neck when grazing, but loose enough so that the cow was comfortable. The VF device was positioned on the top of the neck. After collar attachment, cows were released into the home pen to allow habituation to the collars for 2 days. During habituation, the collars were inactive. Therefore, no stimuli were delivered. For the duration of the habituation period, cattle were housed in a large 100 m × 80 m paddock, the “home pen”, adjacent to the test site with access to shade and water. Animals were offered a maintenance ration of Lucerne hay (dry matter 89%, crude protein 16.4%, metabolizable energy 8.5 MJ ME/kg DM). The maintenance energy requirement for the herd was estimated as per Corbett et al. [18] and all cows were provided ad libitum access to water via a water trough. 

### 2.2. Experimental Paddock

**Figure 1:**
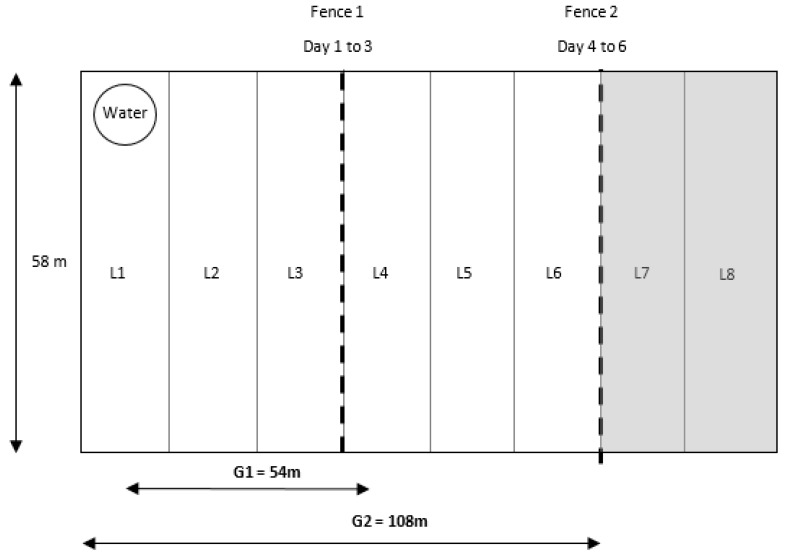
The training paddock used to evaluate learning of the virtual fence stimuli and spatial usage of the paddock. The position of the water trough is indicated. The paddock was divided into 8 equal locations (L1 to L8 as indicated) using GPS co-ordinates, as indicated by the dashed lines. On days 1 to 3, cows were offered L1 to L3 (grazing allocation 1, G1), and on days 4 to 6, cows were offered L1 to L6 (grazing allocation 2, G2). A third section of pasture (grey shaded area) remained unallocated as the exclusion zone. A virtual fence was set at L3/L4 for G1 and L5/L6 for G2, as indicated by the dashed lines.

After the habituation period, the experiment was conducted over 6 days, with cows offered two 3-day grazing allocations of pasture (G1 and G2). Animals were offered a maintenance ration of irrigated annual ryegrass with energy requirements determined as per Corbett et al. [18]. A rising plate meter (Farmworks, Palmerston North, New Zealand) was used to determine pasture on offer. Nine quadrats (0.25 m^2^) were cut to ground level and dried at 60 °C for 48 h to calibrate the rising plate meter before the experimental period. All cows were provided ad libitum access to a water trough (Figure 1). The total paddock area was 8307 m^2^ (58 m × 144 m), which was divided into 8 equal locations (L1 to L8) of 18 m according to GPS co-ordinates to determine location of stimuli and the subsequent spatial usage of cows in the virtual paddock (Figure 1). Pasture was allocated by VF in two grazing allocations (G1 and G2) of 2769 m^2^. A third section of pasture (G3; 2769 m^2^) remained unallocated (L7 and L8). The virtual fence was positioned within L3 in G1, and within L6 in G2. At 1000 h on day 1, cows were moved onto the experimental paddock, and the VF was activated in G1 to allow cows access to L1 to L3. At 1000 h on day 4, the VF was deactivated in G1 and activated in G2 for a further 3 days to provide the cows access to L1 to L6. The VF activation took a maximum of 15 min for all cows. Therefore, cows spent approximately 72 h (±15 min) within each grazing allocation. At 1000 h on day 6, the VF was deactivated, and cows were removed from the experimental paddock.

### 2.3. Virtual Fence Collars

Virtual fence collar prototypes have been described previously [13]. For this experiment, cows were fitted with an experimental prototype automated virtual fencing collar (eShepherd™, Agersens Pty Ltd., Melbourne, VIC, Australia). The virtual fencing experimental prototype, as provided by Agersens, consisted of a strap and hanging counterweight (total weight approximately 1.4 kg) and a unit (approximately 725 g and 17 cm L × 12 cm W × 19 cm H), positioned on the top of each animal’s neck. Using GPS technology, the unit monitored the animal’s movement and provided a real-time measure of the animal’s position, heading and speed. A VF boundary separating inclusion versus exclusion zones, specified using GPS coordinates, was transmitted to the unit using a radio frequency link. As the animal approached the virtual fence boundary the unit emitted a distinctive audio tone within the animal’s hearing range. If the animal stood still or turned away, no electrical stimulus was applied. If the animal continued to move through the virtual fence boundary into the exclusion zone, the unit delivered a short, sharp pulse in the kilovolt range (values are commercial in confidence). This sequence of an audio tone followed by the electrical pulse was repeated if the animal continued through the fence line and into the “exclusion zone”. No stimuli were applied if the animal turned around to stay within the inclusion zone. If movement was above or below a specified velocity (values are commercial in confidence), stimuli were not applied. If an individual animal received a specified number of stimuli within a specified time frame, the device entered standby mode and stimuli were not applied for a specified time frame (values are commercial in confidence, Agersens, VIC, Australia). The collar also included a grazing algorithm whereby if an animal gradually encroached on the exclusion zone by grazing (slow movement forward paired with stopping), an electrical pulse was applied after 3 consecutive audio tones. The grazing algorithm is unidentifiable in the data sets and was therefore not accounted for in the analysis. The date, time, GPS location and “event” which included where the cow was in relation to the inclusion zone and details of stimuli delivery were logged for later download from the unit.

### 2.4. Data Processing

Data from the collars of each cow was downloaded, and the date- and time-stamped GPS location and stimuli details (audio tone and electrical pulse) were recorded for the study.

Collar GPS data for each cow was downloaded in csv. format and data points outside of paddock boundaries were removed (0.5% of total). A total of 647,249 location points were used in the final analysis. The average time spent within each of the set locations was calculated as a proportion of total time per day within each grazing allocation. In addition, the number of audio tones and electrical pulses delivered to each individual cow was calculated for each day of grazing.

### 2.5. Statistical Analysis

A restricted maximum likelihood approach (REML) in Genstat (Version 17, VNSi, UK) was used to analyse the proportion of time spent grazing, and the number of audio tones and electrical stimuli delivered. As different locations were offered for each grazing allocation, the proportion of time cows spent in each location within G1 and G2 were analysed separately. For each proportion of time spent grazing, the interaction between the fixed effects of day of grazing within each allocation (day 1 to 6) and location (L1 to L6) was tested with CowID included as the random effect. The interaction between CowID and location was analysed for each grazing allocation (G1 and G2), with day of grazing included as the random effect. For significant interactions, post-hoc pairwise comparisons were performed using least significant differences for the model-derived predicted means.

The number of audio tones and electrical pulses were compared between grazing allocations and across days of grazing, with CowID included as the random effect. As cows were provided access to different locations for each grazing allocation, data was restricted to each grazing allocation to test the effect of location. The difference in number of each stimuli delivered to individual cows (CowID) was tested, with day of grazing included as the random effect.

Significance was determined at *p* < 0.05. A statistical tendency was considered at 0.05 < *p* < 0.1.

## 3. Results

### 3.1. Proportion of Time Grazing

The results are presented in Table 1. For both G1 and G2, there was a significant interaction between location and day of grazing (*p* < 0.001). In both grazing allocations, cows spent less than 1% of their time in the exclusion zone each day. The amount of time cows spent grazing in the exclusion zone was similar across days. In G1, cows were in L1 for the greatest proportion of time on days 1 and 3, and were in L3, near the VF, for the least proportion of time across all days. The proportion of time cows were in L3 increased from day 1 to days 2 and 3.

In G2, cows spent the greatest proportion of time in L4, the middle of the allocation, across all days. Cows spent more time in L1 on day 4 than on days 5 and 6. There was no difference between days for the time spent in L5 and L6.

The proportion of time spent in each location varied between cattle. For each grazing allocation, there was a significant CowID x Location interaction (Table 2, *p* < 0.001). Cow 8 spent the most time grazing near the VF in both grazing allocations.

### 3.2. Stimuli Delivery

The number of stimuli delivered per day reduced from G1 to G2 (*p* < 0.001, Table 3). The proportion of electrical pulses to audio tones halved between G1 (20%) and G2 (12%).

There was a reduction in the mean number of audio tones and electrical pulses from day 1 to day 4 of grazing (*p* ≤ 0.01, Table 4). The ratio of EP:AT was greatest on day 1 of the experimental period.

In G1, the number of audio tones delivered differed between locations (Table 5) (*p* < 0.001). The greatest number of audio tones were delivered in L3, which was the location of the VF for G1. Electrical pulse delivery was similar for each location. In G2, stimuli were only delivered in L6.

The number of audio tones and electrical pulses delivered to individual cows varied (Table 6). Cows 8 and 12 received the greatest number of stimuli with cow 12 receiving more than double mean audio tone (AT) and electrica; pulse (EP) as compared to cows 1 to 7 and 9 to 11. There was no difference in EP:AT between cows.

## 4. Discussion

Our findings present new and important results as to how individual dairy cows learn and respond to a virtual fence in an intensive grazing setting. Our work shows that virtual fences can contain dairy cows highly effectively (99% of time) within their allocated area. In both grazing allocations, the herd spent less than 1% of time in the exclusion zone across the 6 days of grazing. This is significantly less than that reported in a previous study where cows spent greater than 10% of their time in the exclusion zone within the initial 48 h period [9]. However, this study used an earlier prototype of the virtual fence collars, and reported malfunction of five of the 11 collars which resulted in the removal of animals from the experiment [9]. In addition, there was evidence of associative learning in our experiment, as demonstrated by a significant reduction in the number of electrical pulses and proportion of EP:AT delivered between the first and second grazing allocations. However, there was significant variation in how individual cows responded to the virtual fence as evidenced by time spent in sections across the paddock, and the quantity of stimuli they received. Thus, cow individuality should be considered when implementing this technology across a herd to minimise the number of electrical pulses received.

The area closest to the fence was underutilised as compared to other locations within the inclusion zones. In G1 the herd spent the least amount of time near the fence in L3 across all three days of grazing, and in G2, less time in L6 as compared to L3, L4 and L5 (Table 1). This may indicate an aversion to the stimuli. A previous study demonstrated that cattle trained to a virtual fence barrier showed greater avoidance of the location where aversive stimuli were received than those trained to an electric fence [14]. Another study investigating the effect of an electric fence on the grazing behaviour of cows demonstrated that cows in paddocks without electric fences performed more grazing behaviour, and grazed closer to the fence, than cows in paddocks with electric fences [19]. In the current study, the electrical pulse and memory of the location in which this occurred, may have impacted spatial usage of the paddock. However, the amount of time cows grazed in L3 increased over time in G1, with the proportion of time in L3 doubling in days 2 and 3 as compared to day 1. This may reflect learning of the stimuli as noted above, or alternately may be a result of depletion in pasture across days of grazing, creating motivation for cows to graze closer to the VF. Future studies should include more detailed pasture measurements to test this hypothesis. In G1, cows received stimuli at the fence line in L3, and into the exclusion zone in L4 to L6, as compared to G2 where cows only received stimuli at the fence line in L6. In G1, cows were learning to pair the stimuli, and were potentially running forward into the exclusion zone, as reported in an earlier study using similar technology [10]. This raises the question as to the impact on the affective state of the animals, where the motivation to access fresh pasture may be competing with aversion to the stimuli. As a system, VF appears successful at a herd level. However, the success of VF technology at an individual animal level relies on animals associating the audio tone with the electrical pulse, thereby learning to respond to the audio tone alone, to remain within an inclusion zone [5].

We showed that cattle learned to associate the audio tone with the electrical pulse, as evidenced by the significant reduction in the mean number of electrical pulses, and the ratio of EP:AT delivered in G2 as compared to G1. The proportion of electrical pulse to audio tones was the greatest on Day 1, almost double that of the successive 5 days of grazing. This provides strong evidence that cows learn to respond more to the audio tone alone than the paired stimuli after the first day of grazing. These results in dairy cows align with previous research investigating the response of Angus heifers to virtual fences in a grazing setting, which reported an increase in the proportion of audio-only responses with time [9,13]. There was a reduction in interactions with the fence in G2, as reflected by lower number of total stimuli, which is likely due to the larger allocation of space (75% of the paddock) in G2 as compared to G1 (37.5%). More space in G2 may have reduced pressure on the VF, which may make it easier for cattle to turn away from the stimulus rather than run forward to escape it [9]. Stocking rate has been shown to reduce grazing behaviour and increase agonistic interactions as cows compete for access to resources [19]. Optimal stocking rate was not reported in the current study; however, this should be evaluated in future longitudinal work. On a herd level, the response to VF stimuli is consistent with expectations for the technology. However, in the current study, there was significant inter-individual variation in paddock utilisation and the number of stimuli delivered.

For both grazing allocations, there were individual cows that grazed closer to the fence than others. For example, Cow 8 spent the most time grazing near the fence in both G1 (L3) and G2 (L6) (Table 2), and received a greater number of audio tones and electrical pulses than most other cows (Table 6). Cow 12 was similar, with the second greatest proportion of time grazing near the fence in G1. However, this did not carry over into G2. Furthermore, Cow 12 received the greatest mean number of both AT and EP across the study. Those animals that have aversive responses to stimuli may not graze as efficiently throughout the paddock, avoiding the fenceline, which could affect production outcomes. This reinforces the need to measure the physiological and welfare impacts of this technology on an individual level. It is therefore important to consider not only the difference in how individuals use the space within a virtual paddock, but how they learn and respond to stimuli.

While over half of the cows in the study received less than two EP per day on average, there was significant variation in the number of EP received between individuals, and the proportion of EP:AT. Individual variation in response to VF stimuli has been consistently reported in Angus heifers, where some animals learned the paired stimuli quickly, while others received electrical pulses on most interactions with the VF [5,9,10,13]. The number of EP delivered to individual cows is important, as there is ongoing debate as to the acceptability of the use of electrical stimuli for animal welfare reasons [8]. However, the use of EP is the most effective form of negative reinforcement for virtual fencing systems to date [5,8]. In the current study, one-third of the cows in the study received greater than three EPs per day across the 6 days of grazing. The acceptable level of stimuli is currently undetermined due to limited literature on the welfare implications of this technology in longer-term grazing settings [18]. In conventional electric fence systems, cattle have been demonstrated to learn quickly, with the majority of electrical pulses received within the first day of learning, and very few electrical pulses thereafter [20]. Cattle received up to seven electrical pulses within the first half day of containment in a small area during training to an electric fence. However, in the 7 days following training, only one cow out of 19 received one electrical pulse [21]. Untrained cattle in the same experiment received only 1 to 2 electrical pulses on the first day and, by day 2, none of the animals made contact with the fence [21]. The aim of VF systems is to achieve the same response. However, the systems have not been directly compared in the literature. In the current experiment, EP were being delivered up to day 6. Associative learning is reflected in a reduced EP:AT. Therefore, while learning appears to happen on a herd level as reflected by overall reduction in EP:AT, when broken down into individual response, the question arises as to whether all cows are learning effectively if some are still receiving EP. It is well accepted that dairy cows, as with all domestic farm animals, show consistent individual variation in the way they respond to environmental challenges [16]. The consequence of individual cows failing to learn the association of stimuli is helplessness, which has significant implications for animal welfare [17]. The current study, and previous research has only investigated responses over a short period of time. Previous studies have reported minimal impact of virtual fence collars on behaviour [9], and similar short-term behavioural, endocrine and physiological responses to those of cattle restrained in a crush [22]. There is a need to determine whether response and learning continues to improve and persists over longer durations of grazing to ensure there is no detrimental impact on animal welfare. Measures of stress were not included in the current study; therefore, it is important that future studies evaluate the impact of the technology on welfare. This consideration is essential when implementing new technologies that have the potential to manipulate animal behaviour [17]. It may be the case that these systems do not work for all individuals within a herd, as indicated by the individual variation between spatial usage and stimuli delivery. Future studies evaluating the physiological and welfare outcomes within these systems will inform recommendations around how a VF system is implemented to ensure optimal outcomes for both the animal and the producer.

## 5. Conclusions

This research has demonstrated that an experimental prototype, automated virtual fence collar was highly successful at containing dairy cows within a grazing allocation. Animals were able to respond increasingly to an audio tone alone. However, there was a high degree of variation between individuals in this response which may affect the efficient use of the paddock. The success of VF technology should not simply be based on animals remaining within an inclusion zone. A more accurate depiction of learning is reflected as a reduction in the number of electrical pulses delivered with time, with the ultimate aim of an animal responding to the audio tone alone, through associative learning, to remain within the inclusion zone. Future research should evaluate how the trend for stimuli delivery continues through time, and how this applies in a variety of grazing environments. There is a need to measure the effect of this technology on individual animal welfare, over longer durations of grazing, to determine the optimal implementation of the system for all individuals within a herd.

## Figures and Tables

**Table 1:** Model-predicted means for the proportion of time spent grazing in locations 1 to 8, across three days of grazing within each grazing allocation G1 and G2. Number of degrees of freedom (n.d.f.) and standard error of the difference (SED) are presented. A virtual fence (VF) line was set at the boundary of L3 for G1 and L6 for G2. Means with different superscripts are significantly different. ^ABC^indicates significant difference between days, within a location; ^a–f^ indicates significant difference between locations, within a day.

Grazing Allocation	Location	Day 1	Day 2	Day 3	n.d.f.	SED	*p*-Value
G1	L1	44.27 ^Aa^	34.29 ^Ba^	40.39 ^Ca^	14	1.63	<0.001
	L2	35.63 ^Ab^	39.15 ^Bb^	32.66 ^Ab^			
	L3	10.84 ^Ac^	22.43 ^Bc^	23.32 ^Bc^			
	L4	0.15 ^d^	0.09 ^d^	0.03 ^d^			
	L5	0.36 ^d^	0.03 ^d^	0.01 ^d^			
	L6	0.34 ^d^	0.01 ^d^	0.01 ^d^			
	L7	0 ^d^	0.01 ^d^	0 ^d^			
	L8	0 ^d^	0 ^d^	0 ^d^			
		**Day 4**	**Day 5**	**Day 6**			
G2	L1	11.84 ^Aa^	4.96 ^Ba^	6.72 ^Bad^	16	1.89	<0.001
	L2	5.49 ^Ab^	0.85 ^Bb^	3.99 ^ABaf^			
	L3	16.07 ^Ac^	21.37 ^Bc^	23.81 ^Bb^			
	L4	34.34 ^Ad^	38.21 ^Bd^	26.47 ^Cb^			
	L5	24.01 ^e^	23.45 ^c^	23.2 ^b^			
	L6	5.58 ^b^	9.11 ^e^	8.99 ^d^			
	L7	0.01 ^f^	0.01 ^b^	0.1 ^e^			
	L8	0 ^f^	0 ^b^	0.62 ^ef^			

**Table 2:** Variation in the proportion of time individual cows were in locations L1 to L8 in grazing allocations G1 and G2 with standard error of the differences (SED). ^A–E^ For each cow, different superscripts denote significant difference between locations.

Grazing Allocation	CowID	L1	L2	L3	L4	L5	L6	L7	L8	n.d.f.	SED	*p*-Value
G1	1	42.93 ^A^	32.41 ^Baef^	14.83 ^C^	0.03 ^D^	1.14 ^D^	1.06 ^D^	0 ^D^	0 ^D^	88	3.16	<0.001
	2	35.05 ^A^	37.66 ^A^	25.49 ^B^	0.01 ^C^	0 ^C^	0.03 ^C^	0 ^C^	0 ^C^			
	3	21.11 ^A^	50.8 ^B^	24.71 ^A^	0.01 ^D^	0 ^D^	0.02 ^D^	0 ^D^	0 ^D^			
	4	49.06 ^A^	26.47 ^Bbe^	19.26 ^C^	0.21 ^D^	0.18 ^D^	0.18 ^D^	0.03 ^D^	0 ^D^			
	5	49.52 ^A^	35.95 ^B^	7.49 ^C^	0.14 ^D^	0 ^D^	0.02 ^D^	0 ^D^	0 ^D^			
	6	45.62 ^A^	30.71 ^B^	20.08 ^C^	0.05 ^D^	0 ^D^	0.03 ^D^	0 ^D^	0 ^D^			
	7	43.85 ^A^	26.17 ^B^	18.78 ^C^	0.01 ^D^	0.04 ^D^	0.01 ^D^	0 ^D^	0 ^D^			
	8	19.63 ^A^	37.96 ^B^	39.51 ^B^	0.06 ^C^	0 ^C^	0.05 ^C^	0 ^C^	0 ^C^			
	9	30.7 ^A^	50.58	15.15 ^C^	0.15 ^D^	0.03 ^D^	0 ^D^	0 ^D^	0 ^D^			
	10	43.7 ^A^	47.71 ^Ad^	5.37 ^C^	0.07 ^D^	0.08 ^D^	0.03 ^D^	0 ^D^	0 ^D^			
	11	49.88 ^A^	33.25 ^B^	8.62 ^C^	0.18 ^D^	0.22 ^D^	0.04 ^D^	0 ^D^	0 ^D^			
	12	45	21.34 ^B^	25.68 ^C^	0.22 ^D^	0 ^D^	0.05 ^D^	0.01 ^D^	0 ^D^			
G2	1	10.13 ^A^	1.91 ^B^	22.13 ^C^	41.02 ^D^	12.62 ^A^	8.12 ^A^	0.03 ^B^	0.44 ^B^	88	3.69	<0.001
	2	8.54 ^AE^	0.59 ^B^	20.95 ^C^	36.04 ^D^	17.9 ^C^	13.19 ^CE^	0 ^B^	0 ^B^			
	3	9.99 ^A^	0.87 ^B^	22.3 ^C^	38.01 ^D^	21.68 ^C^	3.52 ^AB^	0 ^B^	0 ^B^			
	4	7.47 ^AE^	4.44 ^AE^	21.47 ^B^	31.52 ^C^	20.18 ^B^	9.75 ^E^	0.02 ^A^	0.02 ^A^			
	5	8.24 ^AB^	3.65 ^AE^	11.35 ^B^	20.33 ^C^	47.04 ^D^	6.78 ^ABE^	0 ^E^	0.45 ^E^			
	6	20.22 ^A^	2.52 ^BE^	9.56 ^BC^	21.29 ^A^	30.66 ^D^	12.29 ^C^	0 ^E^	0.19 ^E^			
	7	16.38 ^A^	1.5 ^BE^	7.67 ^B^	37.56 ^C^	24.85 ^D^	8.22 ^B^	0.17 ^E^	0.13 ^E^			
	8	2.3 ^AE^	8.27 ^AB^	15.28 ^BD^	33.42 ^C^	20.43 ^D^	15.49 ^BD^	0.05 ^E^	0.04 ^E^			
	9	0.78 ^A^	1.58 ^A^	27.54 ^B^	40.71 ^C^	24.66 ^B^	1.74 ^A^	0 ^A^	0 ^A^			
	10	0.91 ^A^	0.85 ^A^	37.02 ^B^	38.34 ^B^	16.57 ^C^	2.18 ^A^	0 ^A^	0.42 ^A^			
	11	4.26 ^AE^	11.36 ^A^	18.93 ^B^	26.34 ^C^	27.34 ^C^	7.88 ^A^	0.16 ^E^	0.3 ^E^			
	12	5.89 ^A^	3.15 ^AE^	27.85 ^B^	36.4 ^C^	18.97 ^D^	4.96 ^A^	0.03 ^A^	0.21 ^A^			

**Table 3:** Mean number of stimuli delivered per animal per day across the 3 days of each allocation G1 and G2. Number of degrees of freedom (n.d.f.) and standard error of the difference (SED) are presented. * indicates significant difference between means of the same stimuli type.

Stimuli		G1	G2	n.d.f	SED	*p*-Value
Audio Tone:	Mean	17.2	9.5 *	1	1.8	<0.001
Electrical Pulse	Mean	3.4	1.5 *	1	0.5	<0.001
(EP:AT) Ratio (%)		20	12 *	1	2.9	0.01

**Table 4:** Model-predicted means for number of audio tones and electrical pulses delivered across each day of grazing. Number of degrees of freedom (n.d.f.) and standard error of the difference (SED) are presented. ^a,b,c^ Indicates significant difference in mean number of audio tones delivered between locations.

Stimuli	G1			G2			n.d.f.	SED	*p*-Value
	Day of Grazing								
	1	2	3	4	5	6			
Audio Tone:	14.5 ^ab^	18.7 ^a^	19.3 ^a^	10.4 ^b^	9.8 ^b^	8.3 ^b^	5	3.2	<0.01
Electrical Pulse	4.2 ^a^	2.8 ^abc^	3.1 ^ab^	1.9 ^bc^	1.3 ^c^	1.2 ^c^	5	0.9	<0.01
(EP:AT) Ratio (%)	29^a^	13^b^	16^b^	17^b^	10 ^b^	9^b^	5	4.4	< 0.001

**Table 5:** Predicted mean stimuli and total number of stimuli delivered within locations 3 to 6 for grazing allocation 1. ^a,b^ Indicates significant difference in mean number of audio tones delivered between locations.

Stimuli	L3	L4	L5	L6	n.d.f.	SED	*p*-Value
Audio Tone	15.9 ^a^	2.9 ^b^	7.0 ^ab^	1.4 ^b^	3	5.0	<0.001
Electrical Pulse	2.7	1.4	3.8	1.1	3	1.3	0.09

**Table 6:** Mean number audio tones and electrical pulses delivered, and mean ratio of EP:AT (%) for individual cows per day. Number of degrees of freedom (n.d.f.) and standard error of the difference (SED) are presented. ^abc^ within a grazing allocation, for each type of stimuli, cow responses differ significantly from each other.

CowID	Audio Tone	Electrical Pulse	EP:AT (%)
1	11.7 ^ab^	1.3 ^ab^	11
2	17.5 ^ac^	3.3 ^ab^	18
3	9.0 ^ab^	1.2 ^a^	11
4	11.2 ^ab^	1.8 ^ab^	12
5	6.8 ^b^	1.0 ^a^	10
6	11.7 ^ab^	2.3 ^ab^	23
7	6.3 ^b^	1.3 ^ab^	18
8	26.5 ^cd^	4.0 ^bc^	16
9	6.7 ^b^	1.0 ^a^	11
10	5.0 ^b^	1.0 ^a^	11
11	8.7 ^a^	3.3 ^ab^	26
12	35.8 ^d^	6.3 ^c^	19
n.d.f.	11	11	11
SED	5.1	1.4	7.5
*P*-value	<0.001	0.005	0.42
